# Reduced PGC-1β protein expression may underlie corticosterone inhibition of mitochondrial biogenesis and oxidative phosphorylation in chicken muscles

**DOI:** 10.3389/fphys.2022.989547

**Published:** 2022-10-12

**Authors:** Sheng Li, Zhi Wang, Jing Wen Yao, Hong Chao Jiao, Xiao Juan Wang, Hai Lin, Jing Peng Zhao

**Affiliations:** ^1^ Shandong Provincial Key Laboratory of Animal Biotechnology and Disease Control and Prevention, Key Laboratory of Efficient Utilization of Non-grain Feed Resources (Co-construction by Ministry and Province), Ministry of Agriculture and Rural Affairs, College of Animal Science and Technology, Shandong Agricultural University, Taian, Shandong, China; ^2^ Pharmacy Department, Taian City Central Hospital, Taian, Shandong, China

**Keywords:** corticosterone, PGC-1, mitochondrial integrity, skeletal muscle, broiler chickens

## Abstract

To uncover the molecular mechanism underlying glucocorticoid-induced loss of mitochondrial integrity in skeletal muscles, studies were performed to investigate whether the peroxisome proliferator-activated receptor γ coactivator 1 (**PGC-1**)-mediated pathway was involved in this process. In an *in vivo* trial, 3 groups of 30-d-old Arbor Acres male broilers were randomly subjected to one of the following treatments for 7 days: corticosterone (**CORT**, 30 mg/kg diet), control (blank), and pair-feeding (restricted to the same feed intake as for the CORT treatment), each with 6 replicates of 15 birds. Mitochondrial abundance, morphology, and function were determined in the *pectoralis major* and *biceps femoris* muscles. In an *in vitro* trial, a primary culture of embryonic chick myotubes was incubated with a serum-free medium for 24 h in the presence or absence of CORT (0, 200, and 1,000 nM). Results showed that CORT destroyed mitochondrial ultrastructure (*p* < 0.01), and decreased the enzymatic activity and protein expression of respiratory chain complexes (*p* < 0.05), leading to an inferior coupling efficiency (*p* < 0.05). As reflected by a decline in mitochondrial density (*p* < 0.01) and mitochondrial DNA copy number (*p* < 0.05), CORT reduced mitochondrial contents. Among all three PGC-1 family members, only PGC-1β was down-regulated by CORT at the protein level (*p* < 0.05). Some aspects of these responses were tissue-specific and seemed to result from the depressed feed intake. Overall, CORT may impair mitochondrial biogenesis and oxidative phosphorylation in a PGC-1β-dependent manner in chicken muscles.

## 1 Introduction

Approximately 90% of cellular energies in the form of ATP are generated by mitochondria (Lehninger et al., 1993), whose content and function are associated with performance phenotypes (Kong et al., 2016; Reberter et al., 2017). For example, mitochondria obtained from broilers with lower feed efficiency exhibited greater uncoupling of the electron transport chain (**ETC**) due to site-specific defects, resulting in higher amounts of reactive oxygen species (**ROS**; Ojano-Dirain et al., 2004, 2005; Iqbal et al., 2005; Lassiter et al., 2006; Tinsley et al., 2010). Transcriptional coactivators of the **PGC-1** (peroxisome proliferator-activated receptor γ coactivator 1) gene family, consisting of PGC-1α, PGC-1β, and **PRC** (PGC-1 related coactivator), are master regulators of mitochondrial biogenesis and oxidative phosphorylation (Handschin and Spiegelman, 2006; Liu and Lin, 2011; Villena, 2015). Both PGC-1α and PGC-1β are expressed in tissues with high energy demand including brown fat, skeletal muscle, heart, brain, and kidney, whereas PRC exists ubiquitously (St-Pierre et al., 2003; Lin et al., 2005). Several studies have shown that PGC-1α/β targets the nuclear respiratory factor (**NRF**) system, stimulating the expression of multiple nuclear genes involved in mitochondrial respiration and biogenesis (Wu et al., 1999; Andersson and Scarpulla, 2001; Nisoli et al., 2003; Shao et al., 2010; Wenz, 2013). The current understanding of PGC-1s is mostly based on studies from mammals and little is known whether they function in a similar manner in other types of animals.

Skeletal muscle constitutes 40%–50% of body mass and accounts for 20%–30% of total resting O_2_ consumption (Owen et al., 1978; Zurlo et al., 1990; Dulloo et al., 2010). There have been several reports on the relationship of mitochondrial function and biochemistry with muscle development in broilers (Iqbal et al., 2004; Ojano-Dirain et al., 2007). As the final effector of the hypothalamic-pituitary-adrenal axis, glucocorticoids (**GC**s) help to maintain homeostasis and respond to stressors. If excessive, however, they may impair mitochondrial structure and activity, disrupt muscular energy metabolism, and consequently compromise growth performance and carcass quality (Martens et al., 1991; Marone et al., 1994; Mitsui et al., 2002a; Duclos et al., 2004; Jiao et al., 2018). Since PGC-1s are also likely to play a central role in the transcriptional control of mitochondrial abundance and efficiency in birds, it was hypothesized that the attenuated PGC-1 regulation may contribute to the GC-induced mitochondrial injury.

The aim of the present study was to assess the effect of GCs on the expression of PGC-1 isoforms in chicken muscles that, in turn, influence mitochondrial biogenesis and respiratory function. Using an *in vivo* model with corticosterone (**CORT**)-treated broilers, the protein abundance of PGC-1 family members as well as mitochondrial integrity were characterized in the *pectoralis major* (**PM**) and *biceps femoris* (**BF**) muscles. The results thus obtained were further verified through an *in vitro* experiment with the cultured chick-embryo myotubes.

## 2 Materials and methods

All research procedures were approved by the Animal Care and Use Committee of Shandong Agricultural University and complied with the Regulations on the Administration of Laboratory Animals promulgated by National Science and Technology Commission of the People’s Republic of China (Beijing).

### 2.1 Birds and care

A total of 360 one-day-old male hatchlings (Arbor Acres, *Gallus gallus domesticus*), procured from Shandong Dabao Breeding and Processing Co., Ltd. (Xintai, Taian, Shandong, China), were reared in a 3-tier set of overlap cages (1.40 m × 0.70 m × 0.38 m). Manure was collected on polypropylene belts under each tier of cages and removed out of the house daily from one end. The ambient temperature was 35°C for the first 2 d, then decreased gradually to 22°C at 21 d, and it was maintained as such thereafter. Artificial lighting was continuous before 2 d, and afterward, a 23L:1D schedule was applied. Prior to the commencement of the experiment, all birds received a standard starter (21.0% CP and 3,000 kcal/kg of ME from 1 to 21 d) or grower (19.5% CP and 3,100 kcal/kg of ME from 22 to 29 d) diet. Feed was offered *ad libitum* in mash form and water was available at all times.

### 2.2 *In vivo* corticosterone treatment

At 30 d of age, 270 healthy broilers with similar BW (approximately 1.37 kg) were selected and randomly assigned to 3 groups, each with 2 cages of 15 birds per tier (6 replicate cages per treatment). They were subjected to one of the following treatments for 1 wk: CORT (CAS 50-22-6; ^#^C104537, 98% purity, Aladdin Industrial Corporation, Shanghai, China; 30 mg/kg diet) with free access to feed; blank treatment under *ad libitum* feeding conditions (control); or blank treatment pair-fed to the amount consumed by CORT-administrated chickens. Previous studies have demonstrated that CORT at 30 mg/kg diet was most applicable for investigating its effect in chickens (Malheiros et al., 2003; Lin et al., 2004).

### 2.3 Data collection and sampling

At 37 d of age, feed intake **(FI)** and BW gain were recorded on a per-cage basis to calculate the feed conversion ratio (feed/gain). After a 12-h overnight fast, 2 birds from each cage were randomly selected to represent the average variability of the cage. After weighing, they were euthanatized by cervical dislocation. The breast (both the *pectoralis major* and *minor*) and deboned thigh muscles were harvested and weighed individually, then expressed as a percentage of BW. A 3- to 4-g muscle sample was obtained from the right PM and BF, and immediately frozen in liquid nitrogen for subsequent biochemical analysis and relative quantification of DNA and protein. Portions (around 200 mg) of the left PM and BF muscles were excised for transmission electron microscopy (**TEM**) and mitochondrial function assay.

### 2.4 Additional measurements

#### 2.4.1 Biochemical analysis

The activities of muscle pyruvate dehydrogenase (**PDH**; ^#^BC0385), NAD-dependent malate dehydrogenase (**NAD-MDH**; ^#^BC1040) and citrate synthase (**CS**; ^#^BC1060) were determined spectrophotometrically (Persee T6U, Beijing Purkinje General Instrument Co., Ltd., China) with commercial diagnostic kits (Beijing Solarbio Science and Technology Co., Ltd., China). Also determined were the contents of malondialdehyde (**MDA**; ^#^A003), 8-hydroxy-2′-deoxyguanosine (**8-OHdG**; ^#^H165) and protein carbonyl groups (**PCG**; ^#^A087, Nanjing Jiancheng Bioengineering Institute, China). Total protein concentration of the homogenate was measured by bicinchoninic acid (**BCA**) assay (^#^P0012, Beyotime Biotechnology Co., Ltd., Shanghai, China) using bovine serum albumin as the standard.

#### 2.4.2 Transmission electron microscopy

Muscle samples were trimmed into approximately 1.0 mm^3^ cubes and fixed in 2.5% glutaraldehyde for 24 h at 4°C, followed by 1% osmium tetroxide for 1 h. Then the tissues were dehydrated with a graded series of ethanol solutions, rinsed in propylene oxide, and embedded in epoxy resin (Sihvo et al., 2018; Liaghati et al., 2021). Ultrathin sections (50 nm thick and parallel to the muscle fiber direction) were cut with a PowerTome-XL ultramicrotome (RMC, Boeckeler Instruments, Inc., Tucson, AZ, United States), stained with 2% uranyl acetate and lead citrate, and examined using a transmission electron microscope (JEM-100CX II, JEOL Ltd., Tokyo, Japan) at an accelerating voltage of 80 kV. A minimum of 5 random fields were photographed at 15000× and 30000× magnification from each sample, and the number of mitochondria was counted in a blinded fashion as previously described (Liu et al., 2014).

#### 2.4.3 Mitochondrial function assay

Mitochondria were isolated from the fresh muscle using the Tissue Mitochondria Isolation Kit (^#^C3606, Beyotime) as previously described (Hu et al., 2019). After quantifying the protein content of the mitochondrial suspension, the activities of NADH-coenzyme Q reductase (ETC complex I; ^#^BC0515), succinate-coenzyme Q reductase (complex II; ^#^BC3235), coenzyme Q-cytochrome C reductase (complex III; ^#^BC3245) and cytochrome C oxidase (complex IV; ^#^BC0945) were determined using colorimetric kits (Solarbio). Mitochondrial membrane potential (**MMP**, Δψm) was detected fluorometrically (RF-5301PC, Shimadzu Corporation, Kyoto, Japan) with JC-1 staining (^#^C2006, Beyotime). Mitochondrial oxygen consumption was measured using a Clark-type oxygen electrode (YSI 5300A, Yellow Springs Instruments Co., Ltd., Yellow Springs, OH, United States), and the mitochondrial respiratory control index (**RCI**) was calculated as the ratio of state III to state IV respiration rate.

#### 2.4.4 Mitochondrial DNA copy number

Total DNA was extracted with the SteadyPure Universal Genomic DNA Extraction Kit (^#^AG21009, AG Accurate Biology, Changsha, Hunan, China) and real-time fluorescent quantitative PCR was conducted on the ABI 7500 system (Applied Biosystems, Foster City, CA, United States) with SYBR Green I (^#^04913914001, Roche, Indianapolis, IN, United States), using Mitochondrial DNA (mtDNA) and nuclear DNA-specific primers (Table 1). The final result was represented as *n*-fold differences in gene expression of cytochrome C oxidase subunit III relative to that of the fatty acid synthase, using the control sample as the calibrator (assigned a value of 1).

**TABLE 1:** PCR primers of relevant genes used in this study.

Gene^a^	GenBank no.	Orientation	Primer sequence (5′→3′)	Product length, bp
COX III	NP_006921	Forward	AGG​ATT​CTA​TTT​CAC​AGC​CCT​ACA​AG	71
		Reverse	AGA​CGC​TGT​CAG​CGA​TTG​AGA	
FAS	J03860	Forward	TCCTTGGTGTTCGTGACG	163
		Reverse	CGC​AGT​TTG​TTG​ATG​GTG​AG	

^a^ COX III, cytochrome C oxidase subunit III; FAS, fatty acid synthase.

#### 2.4.5 Western blotting

Frozen muscles were homogenized, lysed, and centrifuged at 12,000 × *g* for 10 min at 4°C. The supernatant was collected, and the protein concentration was determined using a BCA kit (Beyotime). An equal amount (20 μg) of each protein sample was loaded onto 7.5% or 12% SDS-PAGE gels, transferred to polyvinylidene fluoride membranes (Millipore, Billerica, MA, United States of America), and blocked with 5% non-fat dry milk in TBST. The membrane was then incubated with specific primary antibodies overnight at 4°C: PGC-1α (1:1,000; ^#^ab54481, rabbit polyclonal antibody from Abcam, Cambridge, MA, United States), PGC-1β (1:5,000; custom rabbit polyclonal antibody from Wuhan GeneCreate Biological Engineering Co., Ltd., Wuhan, Hubei, China), PRC (1:5,000; custom rabbit polyclonal antibody from GeneCreate), NRF-1 (1:2000; ^#^ab175932, rabbit monoclonal antibody from Abcam), cytochrome C (Cyt C; 1:2000; ^#^ab133504, rabbit monoclonal antibody from Abcam), mitochondrial ATP synthase α-subunit (ATP5A; 1:1,000; ^#^ab176569, rabbit monoclonal antibody from Abcam), glyceraldehyde-3-phosphate dehydrogenase (**GAPDH**; 1:1,000; ^#^AF5009, mouse monoclonal antibody from Beyotime) and β-actin (1:1,000; ^#^AA128, mouse monoclonal antibody from Beyotime). Subsequently, the corresponding secondary antibody (HRP-conjugated goat anti-rabbit or anti-mouse IgG, 1:1,000; Beyotime) was added and incubated at room temperature for 4 h. Chemiluminescence was detected using the BeyoECL Plus kit (^#^P0018S, Beyotime). The band density was normalized to β-actin or GAPDH and presented as fold-change relative to control.

### 2.5 Preparation and culture of chicken embryonic myoblasts

The *pectoralis* muscles were collected aseptically from 15-d chicken embryos and washed twice with D-HANKS solution (^#^H1045, Solarbio). They were minced and digested with 0.1% Pronase E (^#^P8811, Sigma-Aldrich Corp., St. Louis, MO, United States) in high-glucose Dulbecco’s modified Eagle medium (**DMEM-HG**; ^#^SH30243, HyClone Laboratories, Logan, UT, United States) at 37°C for 40 min. Digested cells were then dispersed by repeated pipetting and filtered to remove large debris. The cell suspension was washed twice with D-HANKS and subjected to density gradient centrifugation in discontinuous layers of 20%, 60%, and 90% Percoll (^#^P1644, Sigma). The cell suspension (2–4 × 10^7^ cells/2 ml) was overlaid and centrifuged at 1,500 × *g* for 30–40 min at 8°C. After centrifugation, myoblasts were harvested from the 20%/60% interface, washed twice with D-HANKS, and resuspended in DMEM-HG containing 10% fetal bovine serum (^#^16000-044, Gibco-Invitrogen Corp., Grand Island, NY, United States), 100 IU/ml penicillin and 100 μg/ml streptomycin (^#^15140-122, Gibco-Invitrogen). Myoblasts were seeded in 0.1% gelatin (^#^G1890, Sigma)-coated 6-well plates (Costar ^#^3516, Corning Life Sciences, Acton, MA, United States) at 1 × 10^5^ cells/cm^2^ and cultured for 72 to 96 h at 37°C in a humidified atmosphere of 95% air and 5% CO_2_ until they were differentiated into myotubes. The purity of the myoblast culture was estimated to be >95% by desmin staining with a mouse anti-swine monoclonal antibody (^#^BM0036, Boster Biological Technology Co., Ltd., Wuhan, Hubei, China).

### 2.6 *In vitro* corticosterone treatment

The differentiated myotubes were pre-incubated for 2 h in serum-free DMEM-HG, followed by 24 h incubation in the absence or presence of CORT (0, 200, and 1,000 nM). Each well was considered as one replicate and there were 6 replicates per treatment. Myotubes were then rinsed with D-HANKS, and collected to analyze the protein expression of mitochondrial respiratory chain complexes, mitochondrial coupling efficiency, and protein abundance of PGC-1 family members and NRF-1.

### 2.7 Determination of intracellular ATP

The ATP content in myotube lysates was detected by Bioluminescent Luciferase Assay (^#^S0026, Beyotime) according to the manufacturer’s instruction. Briefly, myotubes were removed with a scraper and collected for centrifugation at 12,000 × *g* for 5 min at 4°C. The pellets were ground with 200 μl lysis buffer and centrifuged again. After protein quantification with BCA Assay (Beyotime), the supernatant was transferred to a new tube and mixed with 100 μl test solution. The emitted light was measured in a luminometer (TD-20/20, Turner Designs Inc., Sunnyvale, CA, United States), and the results were compared to a standard curve of ATP concentrations ranging from 10 to 1,000 nM.

### 2.8 Statistical analysis

All data were subjected to one-way ANOVA using the GLM procedure (SAS Institute, 2004), to evaluate the effect of imposed treatments. Means for the treatments were compared using the Tukey-Kramer test with the appropriate SEM and were considered to be significantly different when *p* < 0.05. A tendency was declared at 0.05 ≤ *p* ≤ 0.10. Percentage and ratio data were subjected to arc-sine transformation before analysis, satisfying the ANOVA requirements for normality and homogeneity of variance. The model was rerun using raw data to obtain least squares means for presentation, but *p*-values were calculated using transformed data.

## 3 Results

### 3.1 Muscle development and metabolism

Compared with the control, CORT decreased (*p* < 0.01) FI, BW gain, and feed conversion efficiency (Table 2). Relative to the pair-fed chickens, the CORT-treated broilers had significantly lower (*p* < 0.01) BW gain and feed conversion efficiency, but similar (*p* > 0.10) breast and thigh muscle percentages of BW.

**TABLE 2:** Effects of corticosterone treatment (CORT, 30 mg/kg diet from 30 to 37 d of age) on growth performance and carcass characteristics of broiler chickens^a^.

Item	Control	CORT	Pair-feeding	Pooled SEM	*p*-value
Growth responses^b^					
Feed intake, g/d/bird	133^a^	121^b^	121^b^	5.46	<0.001
BW gain, g/d/bird	61.6^a^	10.6^c^	50.2^b^	2.43	<0.001
FCR, feed (g)/gain (g)	2.22^b^	12.4^a^	2.48^b^	1.63	<0.001
Carcass composition (37 d)^c^					
Breast muscle yield, %	18.1	17.3	18.5	1.24	NS
Thigh muscle yield, %	14.6	14.8	15.0	0.54	NS

^a-c^Means not sharing a common superscript within a row differ significantly (*p* < 0.05).

^a^ FCR, feed conversion ratio; NS, *p* > 0.10.

^b^ Data are means of 6 replicates of 15 birds per replicate.

^c^ Values are means of 12 individuals of 2 birds per replicate.

**FIGURE 1:**
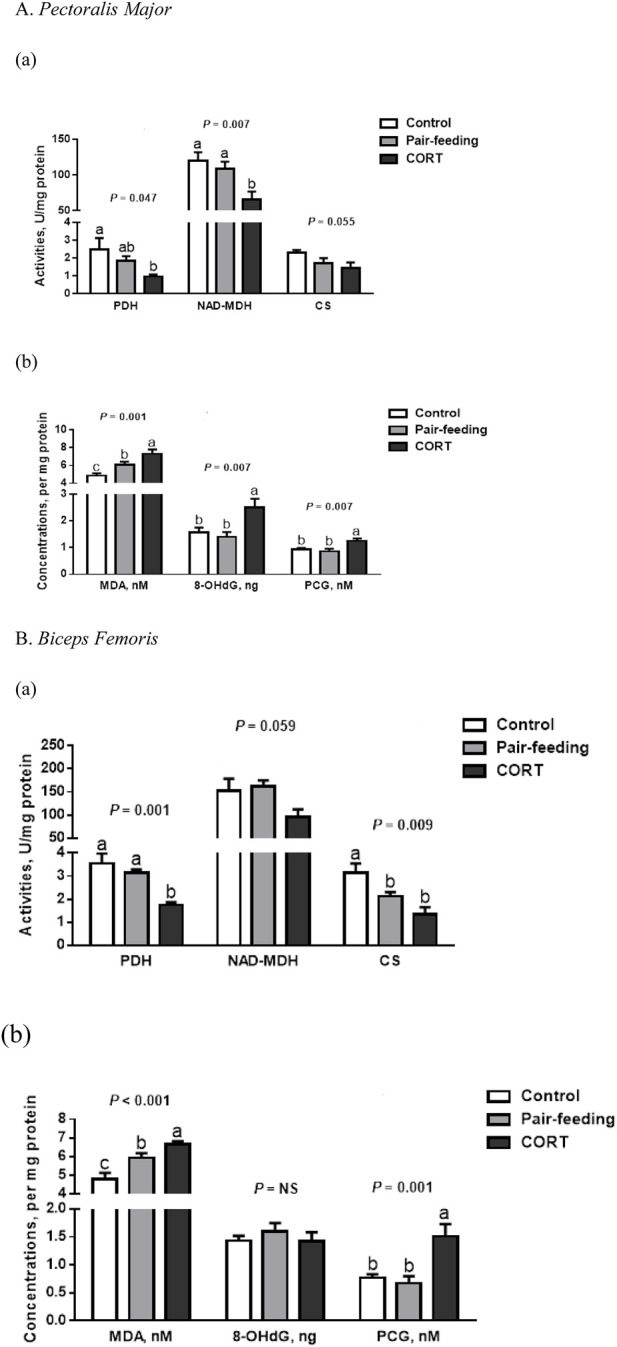
Activities of tricarboxylic acid cycle enzymes **(a)** and concentrations of oxidation products **(b)** in *pectoralis major* **(A)** and *biceps femoris* **(B)** muscles of broiler chickens treated with corticosterone (CORT, 30 mg/kg diet from 30 to 37 d of age). Data are means of 12 individuals of 2 birds per replicate. ^a-c^ Means without a common superscript differ (*p* < 0.05). PDH, pyruvate dehydrogenase; NAD-MDH, NAD-dependent malate dehydrogenase; CS, citrate synthase; MDA, malondialdehyde; 8-OHdG, 8-hydroxy-2′-deoxyguanosine; PCG, protein carbonyl groups; NS, *p* > 0.10.

In comparison to other treatments, activities of NAD-MDH in PM and PDH in BF were all inhibited (*p* < 0.01) by CORT (Figure 1). Likewise, a reduction (*p* < 0.05) in the activities of PDH in PM and CS in BF was observed for the CORT-treated group relative to the control. For CS in PM and NAD-MDH in BF, there was a slight dip (*p* = 0.06) in CORT-exposed birds.

Compared to either control or pair-fed counterparts, birds receiving exogenous CORT showed higher (*p* < 0.01) MDA and PCG formations in both PM and BF (Figure 1). In the PM, the 8-OHdG level was also higher (*p* < 0.01) in CORT-challenged chickens.

### 3.2 Mitochondrial morphology and biogenesis

**FIGURE 2:**
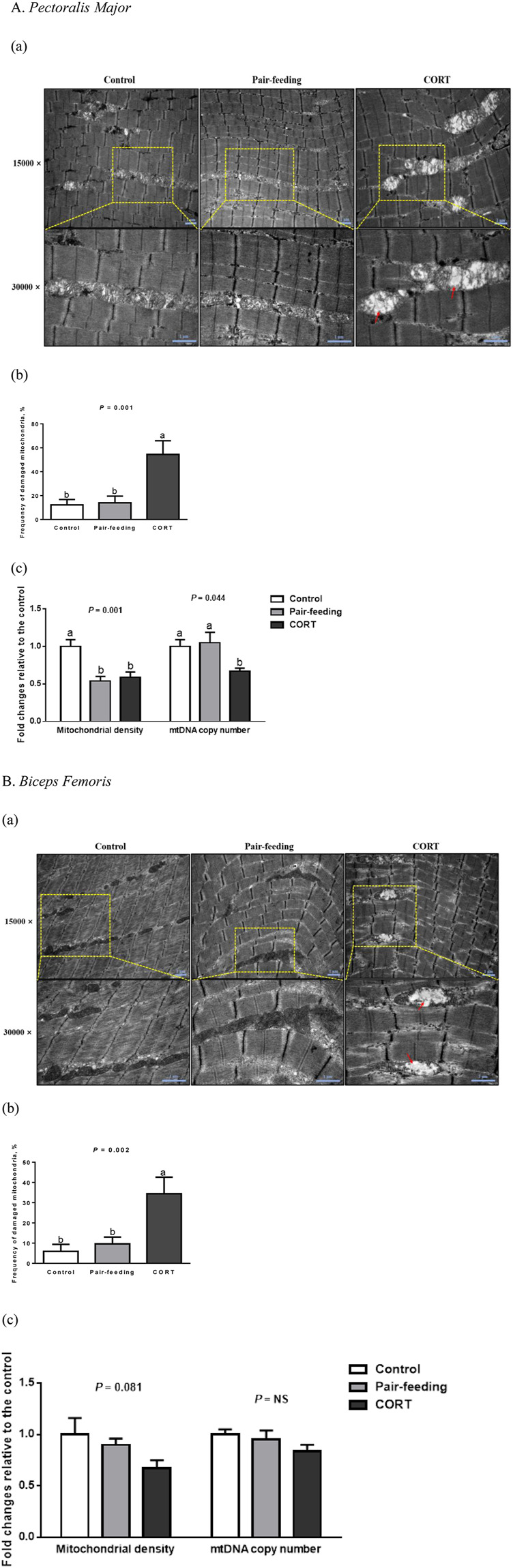
Mitochondrial morphology **(a**, red arrows indicate the ultrastructural injury**)**, frequency of damaged mitochondria **(b)** and mitochondrial quantity **(c)** in *pectoralis major* **(A)** and *biceps femoris* **(B)** muscles of broiler chickens treated with corticosterone (CORT, 30 mg/kg diet from 30 to 37 d of age). Data are means of 12 individuals of 2 birds per replicate. ^a,b^ Means without a common superscript differ (*p* < 0.05). mtDNA, mitochondrial DNA; NS, *p* > 0.10.

In the control and pair-fed groups, the ultrastructural assessment revealed intact mitochondria with clear outer membranes and regular cristae in both skeletal muscles (Figure 2). Conversely, quite a few mitochondria demonstrated visible damages characterized by swelling (increased perimeter and area), vacuolization (decreased cristae density), and crista fragmentation (shortened cristae length) in CORT-exposed birds (*p* < 0.01).

In comparison with the control, a descending trend for mitochondrial density in CORT-administrated birds was significant in PM (*p* < 0.01) and was marginal in BF (*p* = 0.08; Figure 2). In PM but not in BF, CORT decreased (*p* < 0.05) the mtDNA copy number, compared to the control or pair-fed group.

### 3.3 Mitochondrial oxidative phosphorylation

**FIGURE 3:**
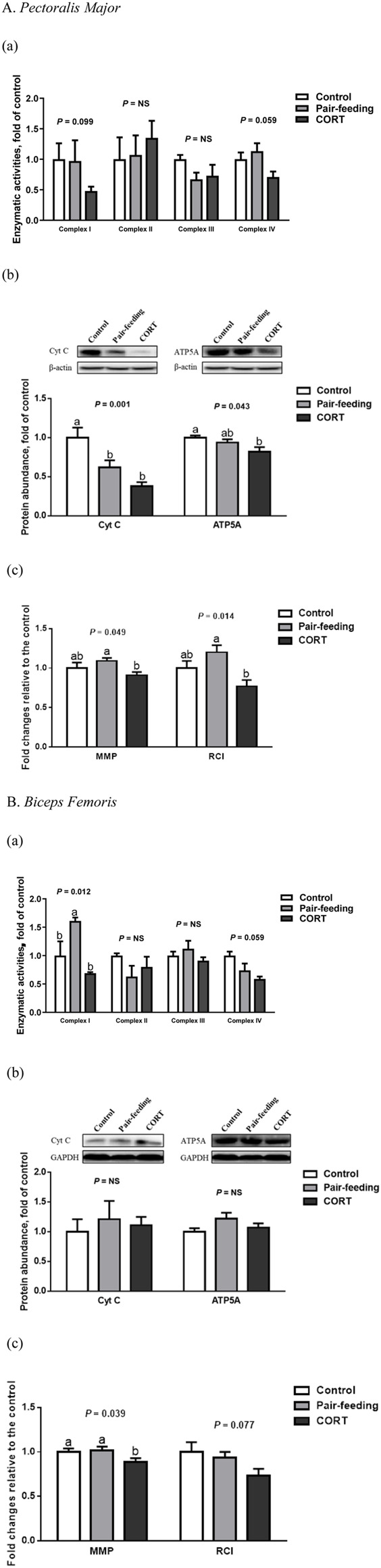
Enzymatic activity **(a)** and protein expression **(b)** of mitochondrial respiratory chain complexes, and mitochondrial coupling efficiency **(c)** in *pectoralis major* **(A)** and *biceps femoris* **(B)** muscles of broiler chickens treated with corticosterone (CORT, 30 mg/kg diet from 30 to 37 d of age). Data are means of 12 individuals of 2 birds per replicate. ^a,b^ Means without a common superscript differ (*p* < 0.05). Cyt C, cytochrome C; ATP5A, mitochondrial ATP synthase α-subunit; RCI, respiratory control index; MMP, mitochondrial membrane potential; NS, *p* > 0.10.

In BF, CORT-administrated broilers had a lower (*p* < 0.05) MMP than did both the control and pair-fed birds (Figure 3). In contrast to the control, CORT decreased the Cyt C (*p* < 0.01) and ATP5A (*p* < 0.05) protein abundance in PM. Compared with the pair-fed subjects, MMP and RCI in PM, and mitochondrial complex I activity in BF were all reduced (*p* < 0.05) in chickens exposed to CORT. Furthermore, mitochondrial complex I (*p* = 0.10) and IV (*p* = 0.06) activities in PM, along with complex IV activity (*p* = 0.06) and RCI (*p* = 0.08) in BF, tended to dwindle under the action of CORT.

**FIGURE 4:**
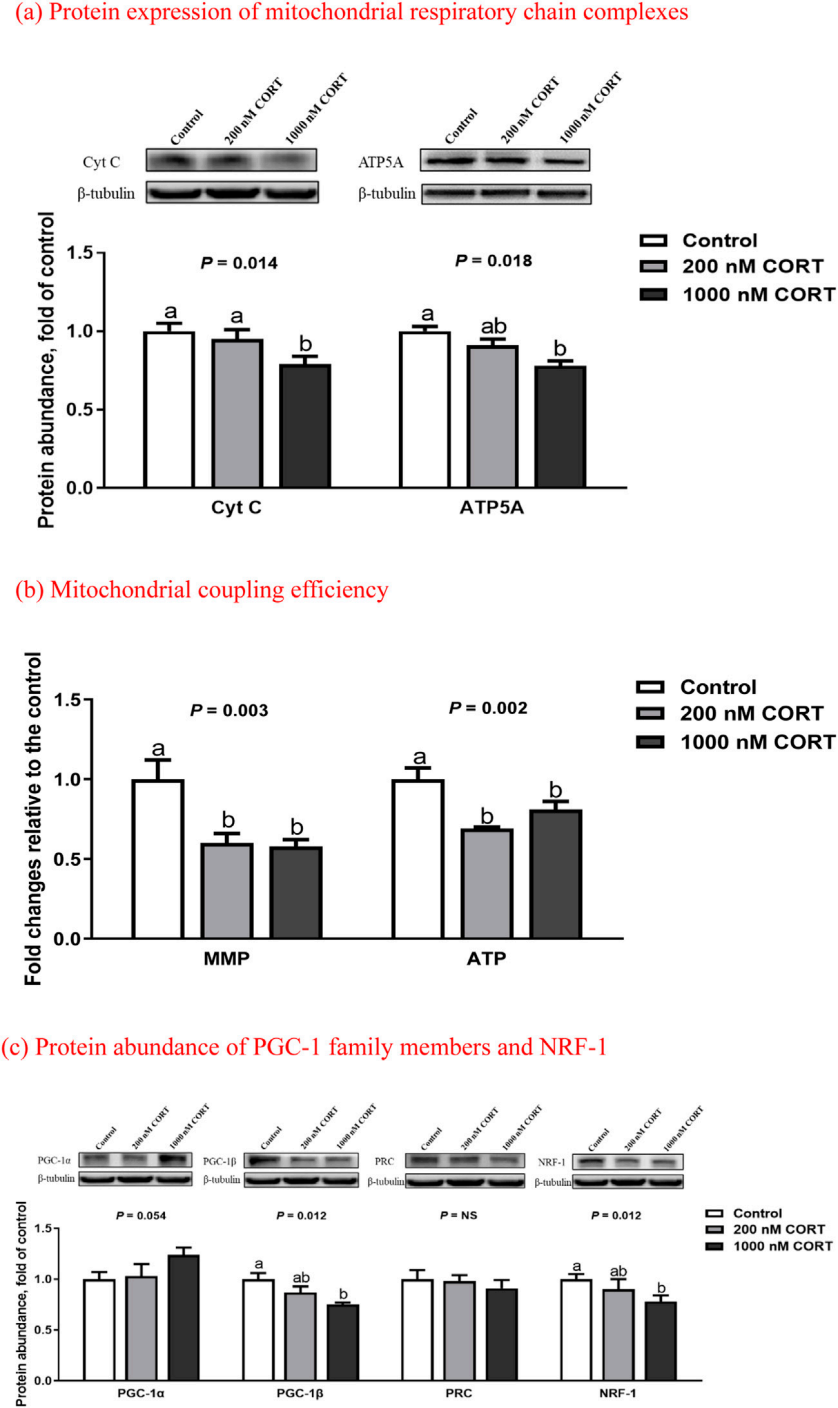
Protein expression of mitochondrial respiratory chain complexes **(a)**, mitochondrial coupling efficiency **(b)**, and protein abundance of PGC-1 family members and NRF-1 **(c)** in embryonic chick myotubes cultured with the indicated concentrations of corticosterone (CORT) for 24 h. Data are means of 6 replicates. ^a,b^ Means without a common superscript differ (*p* < 0.05). Cyt C, cytochrome C; ATP5A, mitochondrial ATP synthase α-subunit; MMP, mitochondrial membrane potential; PGC-1, peroxisome proliferator-activated receptor γ coactivator 1; PRC, PGC-1 related coactivator; NRF-1, nuclear respiratory factor 1; NS, *p* > 0.10.

In cultured myotubes of embryonic chickens, treatment with 1,000 nM CORT suppressed (*p* < 0.05) the protein expression of Cyt C and ATP5A to an abundance less than that of the control (Figure 4). At low and high concentrations of CORT, the levels of MMP and ATP were both decreased (*p* < 0.01), relative to those seen in untreated counterparts.

### 3.4 Protein contents of PGC-1s and NRF-1

**FIGURE 5:**
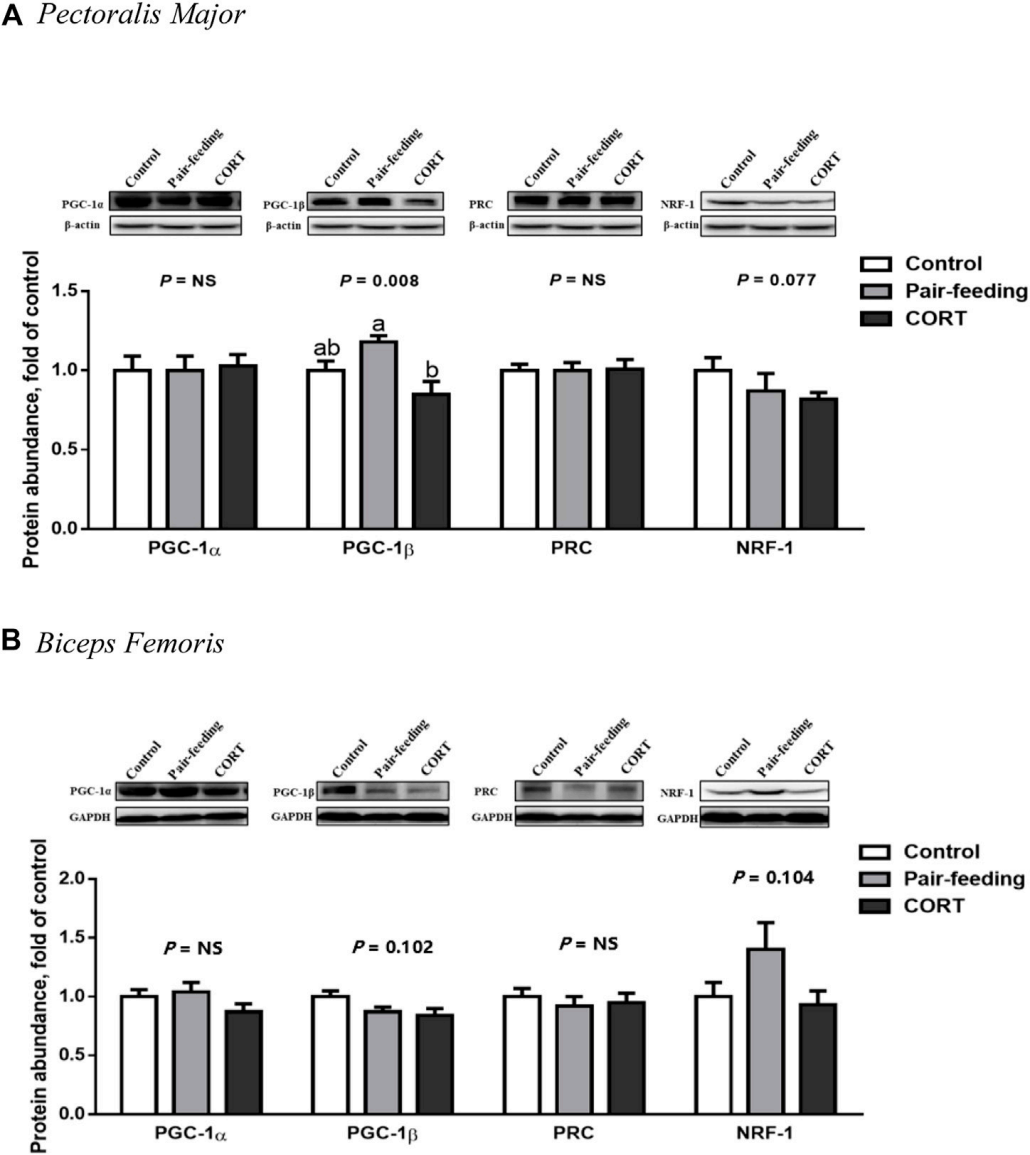
Protein abundance of PGC-1 family members and NRF-1 in *pectoralis major* **(A)** and *biceps femoris* **(B)** muscles of broiler chickens treated with corticosterone (CORT, 30 mg/kg diet from 30 to 37 d of age). Data are means of 12 individuals of 2 birds per replicate. ^a,b^ Means without a common superscript differ (*p* < 0.05). PGC-1, peroxisome proliferator-activated receptor γ coactivator 1; PRC, PGC-1 related coactivator; NRF-1, nuclear respiratory factor 1; GAPDH, glyceraldehyde-3-phosphate dehydrogenase; NS, *p* > 0.10.

Relative to that observed in the pair-fed animals, CORT down-regulated (*p* < 0.01) the protein amount of PGC-1β in PM (Figure 5). The protein expression of PGC-1β in BF (*p* = 0.10) and NRF-1 in both muscles (*p* = 0.08 in PM and *p* = 0.10 in BF) was decreased with the tendency to approach significance in the CORT-treated chickens. No difference (*p* > 0.10) was found in the protein level of PGC-1α and PRC among the 3 treatment groups in either muscle.

In cultured chicken myotubes, the protein abundance of PGC-1β and NRF-1 was reduced (*p* < 0.05) by high-dose CORT exposure (Figure 4), whereas that of PGC-1α was slightly increased (*p* = 0.05). There was no obvious difference (*p* > 0.10) in the expression of PRC between the control and CORT-treated myotubes.

## 4 Discussion

During the period of maximal growth rate for broiler BW and breast weight (Scheuermann et al., 2003), the regulation of muscle mass by exogenous CORT was explored in this study. Although the carcass yield (%) was not affected, the decreased BW gain after CORT administration reflected a drop in the absolute weight of breast and thigh muscles (Song et al., 2011; Zhao et al., 2012). This was consistent with indices of cellular respiration and redox in PM and BF of CORT-challenged birds, including lowered activities of key enzymes in the tricarboxylic acid cycle and increased oxidative damage (Lin et al., 2009; Wang et al., 2010). Considered together with the reduced feed conversion efficiency, failure of the aerobic or oxidative energy system was implicated in CORT-induced muscle atrophy (Vignos and Greene, 1973; Mitsui et al., 2002b).

Mitochondria are vital cellular organelles responsible for energy generation and redox balance. In chickens treated with CORT, a close examination of muscular mitochondria using TEM indicated the presence of abnormal shape and disorganized cristae (Marone et al., 1994; Mitsui et al., 2002b). Coinciding with an increased frequency of damaged mitochondria, relative to the total muscle mitochondrial volume, apparent defects in mitochondrial bioenergetics were noted with CORT exposure. As evidenced by the lowered RCI, CORT increased oxygen consumption to phosphorylate ADP to ATP, resulting in an augmentation of mitochondrial uncoupled respiration (Liu et al., 2016; Shen et al., 2019). Oxidative phosphorylation is a combination of two simultaneous processes, namely the electron transfer and chemiosmotic coupling. Both processes were compromised in chicken muscles following 7 days of CORT manipulation and in cultured embryonic myotubes exposed to CORT for 24 h. On one hand, mitochondrial respiratory chain complexes displayed significant impairment in their activities or protein expressions in response to CORT, which might bring about inefficient electron transport, leading to ROS production (Martens et al., 1991; Mitsui et al., 2002a; Duclos et al., 2004; Jiao et al., 2018). On the other hand, CORT may trigger mitochondrial depolarization and contribute to a decline in ATP synthesis, as a consequence of the dissipation of proton electrochemical potential gradients (collapse of MMP; Troncoso et al., 2014; Chen et al., 2020). Collectively, these findings suggest that CORT treatment predisposes the skeletal muscle to an accumulation of dysfunctional mitochondria involving electron and proton leaks.

Mitochondrial structural and functional integrity is maintained through the coordination of several processes, one of which is mitochondrial biogenesis (Iversen et al., 2011; Picca et al., 2018; Leduc-Gaudet et al., 2021). In the current study, a fall in mitochondrial density and lowered mtDNA copies represented a slowed-down rate of mitochondrial turnover, making the loss of mitochondria a possible biomarker for CORT-mediated muscle atrophy (Liu et al., 2016; Shen et al., 2019). Mitochondrial biogenesis is primarily orchestrated by the PGC-1α/NRF-1/**TFAM** (mitochondrial transcription factor A) axis, which modulates the expression of nuclear and mitochondrial genes that encode mitochondrial proteins (Sandri et al., 2006; Jäger et al., 2007). Although NRF-1 had a mild to marked reduction in protein abundance in chicken muscles with CORT administration under *in vivo* and *in vitro* conditions, PGC-1α expression remained unchanged in both PM and BF muscles, and even appeared slightly up-regulated in cultured myotubes (likely a compensatory response). This response contradicts findings that have been described in rodents exposed to dexamethasone (Qin et al., 2010; Shen et al., 2019). Under stressful circumstances, a PGC-1α-unrelated mechanism of mitochondrial renewal may occur in birds *via* a species-specific phenomenon (Mujahid, 2010; Zhang et al., 2010). Given the concomitant down-regulation of PGC-1β, it is proposed that CORT restrains mitochondriogenesis through a PGC-1β-dependent pathway. The novel finding provides an intervention target to prevent GC-induced mitochondrial dysfunction.

The respiratory chain is composed of 4 complexes (I-IV) embedded in the inner mitochondrial membrane, which are uniquely controlled by mtDNA and the nuclear genome (Calvo et al., 2016). Similar to mammalian mtDNA, chicken mtDNA contains 37 genes, 13 of which encode essential subunits of the oxidative phosphorylation system (complex I, III, IV, and V; Boore, 1999). PGC-1 coactivates NRF to regulate nuclear genes involved in mitochondrial respiration (Shao et al., 2010), while inducing TFAM expression to modulate mtDNA replication and transcription. Both PGC-1α and PGC-1β stimulate mitochondrial biogenesis and functions, but their target genes and pathways are not always the same (Kelly and Scarpulla, 2004). Unlike PGC-1α, PGC-1β is a more potent inducer of mitochondrial respiration and biogenesis. For instance, C2C12 muscle cells expressing PGC-1β had a higher fraction of cell respiration coupled to ATP production than cells expressing PGC-1α (St-Pierre et al., 2003). Moreover, PGC-1α expression activates genes involved in gluconeogenesis (Yoon et al., 2001), whereas PGC-1β expression activates genes involved in β-oxidation of fatty acids (Lin et al., 2003; Ling et al., 2004). As a CORT-responsive PGC-1 in chicken muscles, the specific role of PGC-1β in mitochondrial energy metabolism is currently under investigation through RNA interference and over-expression studies.

It is worth noting that remarkable differences existed between the control and pair-fed groups for several variables in the muscle tissues, suggesting that depressed FI was partially involved in the process of CORT-induced mitochondrial deterioration (Zhang et al., 2020). In addition, PM and BF responded differently to CORT in some aspects, implying a clear tissue specificity. It can be presumed that there are underlying differences between the 2 muscles in stress sensitivity (Lin et al., 2006), perhaps because of the different composition of their myofiber types (Hakamata et al., 2018).

The findings of this study are in disagreement with those of Duan et al. (2018), who evaluated the effects of CORT on mitochondrial metabolism in PM of broiler chickens over a period of 15 days. They failed to see a change in mitochondrial functional status, and reported that the mitochondria were insensitive to exogenous GC. Indeed, the results of these 2 studies are not comparable, due to differences in the delivery route (drinking water vs. feed) and dosage (5 mg/L vs. 30 mg/kg) of CORT. The relationship between GCs and muscle mitochondrial metabolism can be defined as a biphasic response (Duclos et al., 2001; Manoli et al., 2005), that is ineffective or stimulatory/beneficial following moderate exposure (Pouw et al., 2000; Weber et al., 2002; Dumas et al., 2003), but is inhibitory or adverse following excessive exposure (Alesci et al., 2006; Qin et al., 2010).

In conclusion, CORT disrupted the integrity of the mitochondrial structure, quantity, and function, accounting for retarded muscle growth. The diminished protein expression of PGC-1β was likely associated with CORT-suppressed mitochondrial biogenesis and oxidative phosphorylation in chicken muscles. This finding provides new insight for PGC-1β-centered action in GC-induced mitochondrial disorders and suggests a mitochondria-targeted strategy that might prevent muscle atrophy under stressful conditions.

## Data Availability

The original contributions presented in the study are included in the article/supplementary material; further inquiries can be directed to the corresponding author.
